# Genetic Variance in the Adiponutrin Gene Family and Childhood Obesity

**DOI:** 10.1371/journal.pone.0005327

**Published:** 2009-04-24

**Authors:** Lovisa E. Johansson, Lina M. Johansson, Pernilla Danielsson, Svante Norgren, Stina Johansson, Claude Marcus, Martin Ridderstråle

**Affiliations:** 1 Department of Clinical Sciences Malmö, Lund University, Lund, Sweden; 2 National Childhood Obesity Center, Division of Pediatrics, Department of Clinical Science, Intervention, and Technology, Karolinska Institutet, Stockholm, Sweden; Duke University, United States of America

## Abstract

**Aim:**

The adiponutrin gene family consists of five genes (*PNPLA1*-*5*) coding for proteins with both lipolytic and lipogenic properties. *PNPLA3* has previously been associated with adult obesity. Here we investigated the possible association between genetic variants in these genes and childhood and adolescent obesity.

**Methods/Results:**

Polymorphisms in the five genes of the adiponutrin gene family were selected and genotyped using the Sequenom platform in a childhood and adolescent obesity case-control study. Six variants in *PNPLA1* showed association with obesity (rs9380559, rs12212459, rs1467912, rs4713951, rs10947600, and rs12199580, *p*<0.05 after adjustment for age and gender). Three variants in *PNPLA3* showed association with obesity before, but not after, adjustment for age and gender (rs139051, rs12483959, and rs2072907, *p*>0.05). When analyzing these SNPs in relation to phenotypes, two SNPs in the *PNPLA3* gene showed association with insulin sensitivity (rs12483959: β = −0.053, *p* = 0.016, and rs2072907: β = −0.049, *p* = 0.024). No associations were seen for *PNPLA2*, *PNPLA4*, and *PNPLA5*.

**Conclusions:**

Genetic variation in the adiponutrin gene family does not seem to contribute strongly to obesity in children and adolescents. *PNPLA1* exhibited a modest effect on obesity and *PNPLA3* on insulin sensitivity. These data, however, require confirmation in other cohorts and ethnic groups.

## Introduction

A new family of genes with conserved patatin and lipase domains has recently been identified and given the name patatin-like phospholipase family [1]–[3]. The family consists of nine genes, and of these, five genes form a subgroup called the adiponutrin family [3]. This subfamily include patatin-like phospholipase 1 (*PNPLA1*), adipose triglyceride lipase (*ATGL*/*PNPLA2*), adiponutrin (*ADPN*/*PNPLA3*), gene sequence 2 (*PNPLA4*) and GS2-like (*PNPLA5*). It is believed that members of the adiponutrin family complement the hormone sensitive lipase (HSL) as responsible for adipocyte triacylglycerol lipase activity. Mice lacking HSL display a lean phenotype and accumulate diglycerides suggesting that HSL is the main enzyme for the second step of lipolysis and that other enzymes are responsible for the first step [4]–[6]. Several studies indicate that the protein encoded by *PNPLA2* is one of the enzymes responsible for this first step in lipolysis [3], [7]. Less is known about the function of the other members but data indicates that they retain both lipolytic and lipogenic properties [1]–[3].

All members of the adiponutrin gene family are highly expressed in the adipose tissue. Expression increases during adipocyte differentiation and is regulated in by nutritional challenges [1]–[3]. For example, *PNPLA3* is downregulated in the adipose tissue of insulin resistant subjects and upregulated in a glucose dependent fashion in response to insulin stimulation [8]. Two studies have demonstrated genetic association between *PNPLA2* and *PNPLA3* with type 2 diabetes and obesity, respectively [8]–[10]. Far less is known about the other three members of the family, *PNPLA1*, *PNPLA4* and *PNPLA5*. The aim of this study was to investigate the genetic relevance of all five genes in the adiponutrin family in the pathogenesis of childhood obesity and insulin resistance.

## Results

In total, 61 out of 85 selected SNPs were successfully genotyped using the Sequenom platform in a childhood and adolescent obesity case-control material (Table S1 and Figures S1, S2, S3, S4, S5). Clinical characteristics for this cohort have been presented previously and are summarized in Table 1 [11]. Gender distribution was similar between the obese and non-obese children. By definition, the obese group was younger than the normal weight controls (Table 1). The obese subjects all showed a significant degree of insulin resistance (HOMA-IR: 3.04 [2.11–4.49], n = 297).

**Table 1:** Clinical characteristics of the child obesity case-control study.

	Non-obese	Obese	*p*-value
Gender (m/f)	234/257	226/240	0.79
Age (years)	17 (16–18)	13 (10–15)	<0.001
Weight (kg)	61 (55–69)	91 (73–109)	<0.001
Length (m)	1.72 (1.66–1.79)	1.61 (1.51–1.70)	<0.001
BMI (kg/m^2^)	20.7 (19.5–22.3)	34.3 (30.9–38.3)	<0.001
BMI-SDS	0.36 (−0.16–1.03)	5.98 (5.14–7.04)^A^	<0.001

*p*-values are calculated using Wilcoxon Rank Sum test or chi^2^ test for distributions.

A Patients with data available were 463. BMI-SDS – Body Mass Index - Standard Deviation Score.

Logistic regression identified six variants in *PNPLA1* that show association with obesity when adjusting for age and gender (rs9380559, rs12212459, rs1467912, rs4713951, rs10947600 and the coding rs12199580, Table 2 and Table S2). Three variants in *PNPLA3* showed association with obesity (rs139051, rs12483959 and rs2072907, Table 2 and Table S2). This association was unaffected by adjustment for gender but attenuated when adjusting for age (data not shown). No variants in the *PNPLA2*, *PNPLA4* and *PNPLA5* were associated with obesity in this cohort (Table S2).

**Table 2:** Genetic variants in PNPLA1 and PNPLA3 showing significant association with obesity using logistic regression.

Gene	SNP	N (case/control)	MAF (cases)	OR	95% CI	P	*P*_Adjusted_
*PNPLA1*	rs9380559	451/484	0.43	1.42	(1.02–1.98)	0.63	0.038
	rs12212459	440/464	0.41	0.70	(0.50–0.99)	0.94	0.043
	rs1467912	455/482	0.37	0.71	(0.51–1.00)	0.61	0.049
	rs4713951	451/479	0.46	0.71	(0.51–0.98)	0.67	0.037
	rs10947600	456/482	0.41	0.72	(0.52–0.99)	0.87	0.042
	rs12199580	451/485	0.41	0.70	(0.51–0.96)	0.64	0.028
*PNPLA3*	rs139051	453/469	0.35	0.99	(0.71–1.38)	0.014	0.97
	rs12483959	450/484	0.16	0.79	(0.52–1.20)	0.023	0.27
	rs2072907	455/477	0.17	0.82	(0.55–1.24)	0.041	0.35

*p*-values are calculated using Logistic regression including gender and age as covariates, additive model. The presented Odds ratios (OR) are adjusted for age and gender. SNP – Single nucleotide polymorphism, MAF – Minor Allele Frequency, OR – Odds ratio, 95% CI – 95% confidence interval.

The associated SNPs were further analyzed for association with phenotypes related to obesity in the group of obese children and adolescents (Table 3). Similar data for the control group was not available. *PNPLA1* variants rs12212459 and rs1467912 showed association with BMI-standard deviation score (SDS) after adjusting for age and gender (Table 3). Adjusting for insulin resistance defined by HOMA-IR did affect this observation (β = 0.30, *p* = 0.025 and β = 0.40, *p* = 0.0029, respectively). *PNPLA1* SNP rs10947600 was associated with both body weight (n = 330, GG: 104.3[85.1–124.8] kg, GA: 94.1[78.3–111.9] kg, AA: 94.4[79.3–109.0] kg, β = −3.38, *p* = 0.018) and glucose levels (n = 292, GG: 4.9[4.7–5.3] mmol/L, GA: 4.9[4.6–5.2] mmol/L, AA: 4.7[4.5–5.1] mmol/L, β = −0.0077, *p* = 0.032) and *PNPLA1* SNP rs12199580 with glucose levels (n = 289, CC: 4.9[4.7–5.3] mmol/L, CA: 5.0[4.6–5.2] mmol/L, AA: 4.7[4.5–5.1] mmol/L, β = −0.0085, *p* = 0.016).

**Table 3:** Obesity-associated variants in PNPLA1 and PNPLA3 and the association with phenotypes using multiple regression analyses.

SNP		BMI-SDS	Insulin sensitivity (Si*10^−5^/pM/min)	HOMA-IR
**rs9380559**	**n**	323	271	224
	**β**	−0.058	0.0062	−0.15
	***p***	0.60	0.73	0.47
	**AA**	6.07 (5.19–7.20)	0.29 (0.18–0.46)	3.40 (1.99–4.91)
	**AG**	5.61 (4.97–5.68)	0.33 (0.19–0.48)	3.38 (2.46–4.52)
	**GG**	6.03 (5.29–6.71)	0.37 (0.25–0.49)	3.22 (2.35–4.40)
**rs12212459**	**n**	316	268	220
	**β**	0.24	0.0098	0.012
	***p***	0.036	0.58	0.96
	**AA**	5.76 (4.86–6.67)	0.32 (0.19–0.43)	3.44 (2.48–4.61)
	**AC**	5.76 (5.15–6.85)	0.33 (0.19–0.49)	3.38 (2.24–4.90)
	**CC**	6.07 (5.30–7.56)	0.33 (0.21–0.52)	3.30 (1.80–4.48)
**rs1467912**	**n**	329	276	229
	**β**	0.26	0.0048	−0.068
	***p***	0.022	0.80	0.76
	**CC**	5.82 (4.95–6.68)	0.32 (0.19–0.45)	3.36 (2.45–4.61)
	**CT**	5.66 (5.08–6.69)	0.34 (0.20–0.51)	3.35 (2.10–4.74)
	**TT**	6.22 (5.53–7.61)	0.32 (0.21–0.46)	3.43 (2.27–4.63)
**rs4713951**	**n**	326	275	228
	**β**	0.22	−0.014	−0.080
	***p***	0.052	0.45	0.72
	**CC**	5.66 (4.98–6.68)	0.33 (0.20–0.47)	3.30 (2.45–4.19)
	**CT**	5.81 (5.02–6.90)	0.31 (0.18–0.49)	3.64 (2.34–4.83)
	**TT**	6.11 (5.37–7.37)	0.33 (0.22–0.42)	3.19 (1.92–4.53)
**rs10947600**	**n**	327	275	228
	**β**	0.024	0.015	−0.28
	***p***	0.83	0.41	0.20
	**GG**	5.89 (4.97–6.88)	0.33 (0.18–0.48)	3.56 (2.49–5.03)
	**GA**	5.83 (5.12–6.93)	0.33 (0.21–0.48)	3.25 (2.31–4.55)
	**AA**	5.83 (5.02–7.01)	0.28 (0.19–0.45)	3.24 (1.89–4.78)
**rs12199580**	**n**	323	271	224
	**β**	0.078	0.0067	−0.38
	***p***	0.48	0.72	0.088
	**CC**	5.82 (4.92–6.68)	0.34 (0.19–0.49)	3.54 (2.46–5.21)
	**CA**	5.87 (5.14–6.97)	0.33 (0.21–0.47)	3.33 (2.45–4.54)
	**AA**	5.83 (5.14–7.05)	0.28 (0.19–0.45)	3.07 (1.83–4.61)
**rs139051**	**n**	326	274	227
	**β**	−0.060	−0.0223	0.14
	***p***	0.59	0.23	0.46
	**GG**	5.90 (4.98–7.15)	0.33 (0.22–0.50)	3.43 (2.37–4.58)
	**GA**	5.75 (5.00–6.57)	0.33 (0.18–0.47)	3.35 (2.30–4.80)
	**AA**	5.95 (5.14–6.89)	0.30 (0.21–0.42)	3.61 (2.34–4.75)
**rs12483959**	**n**	324	274	228
	**β**	0.029	−0.053	−0.30
	***p***	0.83	0.016	0.25
	**GG**	5.83 (5.00–7.09)	0.33 (0.21–0.49)	3.32 (2.36–4.63)
	**GA**	5.81 (5.05–6.54)	0.33 (0.16–0.42)	3.56 (2.39–5.03)
	**AA**	6.14 (5.28–7.32)	0.34 (0.28–0.65)	2.50 (2.14–4.00)
**rs2072907**	**n**	327	275	229
	**β**	0.034	−0.049	−0.21
	***p***	0.79	0.024	0.40
	**GG**	5.81 (4.99–7.04)	0.33 (0.22–0.49)	3.29 (2.35–4.63)
	**GC**	5.88 (5.02–6.65)	0.27 (0.16–0.42)	3.56 (2.39–4.72)
	**CC**	6.24 (5.47–7.16)	0.33 (0.25–0.64)	2.67 (2.28–4.48)

*p*-values are calculated using multiple regression including gender and age as covariates. SNP – Single nucleotide polymorphism. n – number of observations, β– Regression coefficient, *p* - *p*-value for t-statistic. f – fasting samples, BMI-SDS – Body Mass Index - Standard Deviation Score, HOMA-IR – homeostasis model of assessment - Insulin Resistance.

The obesity associated *PNPLA3* variants rs12483959 and rs2072907 showed association with insulin sensitivity (Table 3) and disposition index (rs12483959: n = 264, GG: 122[66–208], GA: 109[61–214], AA: 74[52–197], β = −0.14, *p* = 0.017 and rs2072907: n = 265, GG: 121[66–209], GC: 109[58–215], CC: 83[56–192], β = −0.12, *p* = 0.043). Adjusting for BMI-SDS did not affect the association with insulin sensitivity (β = −0.053, *p* = 0.015 and β = −0.049, *p* = 0.022, respectively) or disposition index (β = −0.15, *p* = 0.012 and β = −0.12, *p* = 0.031, respectively).

## Discussion

The genetic analysis using TagSNPs in the five genes included in the adiponutrin gene family revealed that some variants within these genes exert a weak but significant effect on obesity in children and adolescents. In this study the association was limited to the two genes *PNPLA1* and *PNPLA3*. Findings concerning *PNPLA3* variants were attenuated when adjusting for age and gender but further analysis indicated that they might influence insulin sensitivity.

Childhood obesity is associated with increased risk of cardiovascular disease [12] and reduced life expectancy [13], [XPATH ERROR: unknown variable "rids-text".]. It is therefore of great importance to study this group in order to identify markers that could recognize individuals predisposed to obesity at an early stage. However, the polygenic nature of obesity makes the search for risk altering genes difficult. Recent studies have identified two strong obesity candidate genes, the fat mass and obesity associated (*FTO*) and melanocortin 4 receptor (*MC4R*) [15]–18. Genes involved in lipid metabolism such as lipases would be relevant to investigate in relation with obesity since it is a state of excessive storage of lipids. In the obese state, the adipose tissue is less efficient in buffering lipids resulting in increased levels in the circulation. These lipids will then be stored in other tissues thereby promoting development of insulin resistance and possibly type 2 diabetes [19]. Genetic studies of for example the important lipase hormone sensitive lipase (*HSL*) show significant associations with measures of obesity suggesting that genes coding for proteins with lipase activity are of importance [20]–[25]. The five adiponutrin gene family members encode proteins that are able to both catalyze the build-up and breakdown of fat thus identifying them as possible candidate genes [1]–[3]. Data presented here, for the most part, failed to clearly confirm this candidacy. We found borderline association with obesity for *PNPLA1* and *PNPLA3*, but these data would not hold for multiple corrections. Given the hypothesis generating nature of the study it is important to underline that the results should be interpreted with caution and need confirmation elsewhere.

So far little is known concerning genetic variation in the adiponutrin gene family and its possible influence on metabolic disease. Only the *PNPLA2* and *PNPLA3* have been studied in this context before. For *PNPLA2*, common variants has been associated with free fatty acid levels, triglyceride levels and type 2 diabetes suggesting that the gene may play an important role for the risk factors associated with obesity rather than obesity *per se* [9]. Rare mutations in the *PNPLA2* gene, resulting in a truncated protein with no capacity to bind to lipid droplets but with an intact patatin domain, has been identified in a subgroup of patients with neutral lipid storage disorder (NLSD) with mild myopathy [26]. NLSD is a disorder characterized by storage of triglyceride-containing cytoplasmic droplets in for example leukocytes, bone marrow, skin and muscle (OMIM #610717). In our study we did not find any association between *PNPLA2* and obesity and therefore no further analysis was conducted. As stated in both previous studies regarding *PNPLA2*, no association was found with obesity and the NLSD patients carrying *PNPLA2* mutations were not obese [9], [26].

Genetic variants in *PNPLA3* have previously been associated with obesity [8]. In this study we confirm these data but also demonstrate an association with insulin sensitivity. The association with obesity disappears when adjusting for age while that with insulin sensitivity association remains. Data may suggest that variants in *PNPLA3* rather affect insulin sensitivity. Although obese adolescents in general are insulin resistant, the degree of obesity is not a major determining factor [27] and together with age, cardiorespiratory fitness, and truncal fat, only 25% of individual variation can be explained [28]. Thus, it is likely that genetic vulnerability is of importance and it is possible that *PNPLA3* variation may play a role It has been shown that both genetic variants and insulin resistance regulate adipose *PNPLA3* gene expression [8], [29].

Genetic variants in the *PNPLA1*, *PNPLA4* and *PNPLA5* genes have not been studied before. We found an association between *PNPLA1* and juvenile obesity but no associations were found for *PNPLA4* or *PNPLA5*. These data need to be replicated due to the relatively small study material used in this study.

In conclusion, although members of the adiponutrin gene family are clear candidate genes for obesity we were unable to clearly confirm this candidacy for obesity in children and adolescents. We did find a modest effect of *PNPLA1* on obesity and *PNPLA3* on insulin sensitivity although these data need confirmatory studies. Furthermore, although *PNPLA2*, *PNPLA4* and *PNPLA5* did not show any significant association with obesity and insulin sensitivity, we cannot rule out a possible implication in the pathogenesis due to the low power of this study.

## Materials and Methods

### Study subjects

We studied 466 obese children and adolescents referred to the National Childhood Obesity Centre at Karolinska University Hospital and Karolinska Institute and 491 non-obese adolescents recruited from 17 upper secondary schools around Stockholm (Table 1) [11]. For the obese children we had available blood samples, growth charts, clinical journal notes, medical examination and laboratory reports as well as questionnaires completed by the parents of the children at enrolment. The lean adolescents were asked through the school nurse if they wanted to participate in the study. Blood was collected and every adolescent completed a questionnaire concerning ethnicity, health and the use of medical drugs. Subjects with overweight/obesity or chronic diseases were excluded from the control group.

Height (Ulmer Stadiometer, Ulm, Germany), and weight (Vetek TI-1200, Väddö, Sweden) were measured with subjects in light clothing and body mass index (BMI; kg/m^2^) calculated. Body weight and height was measured at the first visit to the nearest 0.1 kg and 1 cm, respectively. All subjects in the obese group were obese according to international age and sex adjusted standards [30]. Values of a BMI standard deviation score (BMI-SDS) was calculated from weight, height, age and gender based on a French material from 1982 [31]. All subjects gave their written informed consent and the Regional Committee of Ethics, Stockholm, approved the study. The study was conducted according to the principles of the Helsinki Declaration.

### Laboratory analysis

Blood samples from the obese children for measurement of glucose, (glucose-6-phosphate dehydrogenase method, Kebo Lab, Stockholm, Sweden), insulin (Pharmacia Diagnostics AB, Uppsala, Sweden), HDL-cholesterol and triglycerides (Boehringer, Mannheim, Germany) were obtained after an over-night fast. Analyses were performed at a certified laboratory (Department of Clinical Chemistry, Karolinska University Hospital). The control subjects were not fasting when the blood samples were obtained. Therefore insulin and glucose were measured only in the obese cohort. Insulin resistance was estimated by homeostasis model of assessment (HOMA-IR) [32]. Insulin sensitivity index representing the effect of insulin to catalyze the clearance of glucose from plasma after an intravenous glucose load were calculated using the Bergman minimal model approach [11], [33]. Acute insulin response reflects the first phase of endogenous secretion in response to glucose infusion and was calculated as area under the curve during the first 10 minutes [11], [34]. Genomic DNA was prepared by standard methods. DNA was extracted from whole blood by using QiaGen MaxiPrep (QiaGen, Germany) at the DNA/RNA Genotyping Lab, SWEGENE Resource Center for Profiling Polygenic Disease, Lund University, Malmö University Hospital, Malmö, Sweden.

### Genotyping

SNPs were selected by using data from the HapMap consortium for each of the sequences coding for the selected five genes including an extra 5000 bases upstream and downstream [35]. TagSNPs were then selected using Tagger in the Haploview program for all five genes [36]. Additional coding SNPs were selected from the National Center for Biotechnology Information (NCBI) SNP database (http://www.ncbi.nlm.nih.gov/SNP/). In total, 85 SNPs passed the assay design and were genotyped using the Sequenom platform (MALDI-TOF) at the DNA/RNA Genotyping Lab, SWEGENE Resource Center for Profiling Polygenic Disease, Lund University, Malmö University Hospital, Malmö, Sweden. 24 SNPs failed genotyping and Hardy Weinberg equilibrium. These SNPs were removed from further analysis leaving a total of 61 SNPs for analysis (Table S1 and Figures S1, S2, S3, S4, S5). A selection of SNPs were re-analyzed in a subset of 283 patients using the TaqMan allelic discrimination method on the ABI 7900HT according to manufacturers' recommendations (Applied Biosystems). Success rate was 98.6%.

### Statistical analysis

Logistic regression with age and gender as covariates were used for estimating the genotype association. Linear multiple regressions were performed in order to test for SNP effects on obesity and insulin resistance (HOMA-IR) as quantitative traits. All traits were log transformed for normal distribution. These analyses were adjusted for age and gender. Also, the obesity analysis was adjusted for insulin resistance and *vice versa* insulin resistance analysis for obesity. All *p*-values are based on additive models for the genetic variants. Data are presented as median with interquartile range within brackets [25^th^–75^th^] or odds ratio (OR) with 95% confidence interval (CI). All statistical calculations were performed using PLINK (http://pngu.mgh.harvard.edu/~purcell/plink/index.shtml) [37]. Furthermore, the power to detect an additive OR of 1.2 in this material when the minor allele frequency (MAF) is 0.05 is 17% and 53% for a MAF of 0.5 when α is set at 0.05.

## Supporting Information

Table S1 Hardy Weinberg equilibrium (HWE) for genetic variants analyzed in the Adiponutrin gene family.
(0.12 MB DOC)

Table S2 Variants in the five genes in the Adiponutrin gene family and the association with obesity using logistic regression analyses.
(0.12 MB DOC)

Figure S1 Graphical overview of the patatin-like phospholipase 1 (*PNPLA1*) gene and linkage disequilibrium obtained from HapMap (http://www.hapmap.org/). SNPs successfully genotyped by MALDI-TOF MS are marked with a black square.
(0.62 MB PNG)

Figure S2 Graphical overview of the patatin-like phospholipase 2 (*PNPLA2*) gene and linkage disequilibrium obtained from HapMap (http://www.hapmap.org/). SNPs successfully genotyped by MALDI-TOF MS are marked with a black square. One SNP, the rs1138693, is not included since it was not present in the HapMap database at the time of data extraction. It was included in the study because it is a coding SNP.
(0.01 MB PNG)

Figure S3 Graphical overview of the patatin-like phospholipase 3 (*PNPLA3*) gene and linkage disequilibrium obtained from HapMap (http://www.hapmap.org/). SNPs successfully genotyped by MALDI-TOF MS are marked with a black square.
(0.26 MB PNG)

Figure S4 Graphical overview of the patatin-like phospholipase 4 (*PNPLA4*) gene and linkage disequilibrium obtained from HapMap (http://www.hapmap.org/). SNPs successfully genotyped by MALDI-TOF MS are marked with a black square.
(0.06 MB PNG)

Figure S5 Graphical overview of the patatin-like phospholipase 5 (*PNPLA5*) gene and linkage disequilibrium obtained from HapMap (http://www.hapmap.org/). SNPs successfully genotyped by MALDI-TOF MS are marked with a black square.
(0.17 MB PNG)
