# Supplementary material for: Genetic Variance in the Adiponutrin Gene Family and Childhood Obesity
Source: PLoS One. 2009 Apr 24;4(4):e5327. doi: 10.1371/journal.pone.0005327 (PMC2669125; doi:10.1371/journal.pone.0005327)
Supplement: Table S1 — Hardy Weinberg equilibrium (HWE) for genetic variants analyzed in the Adiponutrin gene family. (0.12 MB DOC) [file pone.0005327.s001.doc]

***Supporting Table S1:*** Hardy Weinberg equilibrium (HWE) for genetic variants analyzed in the Adiponutrin gene family.

|  | **CHR** | **SNP** | **BP** | **Location** | **N(case/control)** | **Minor allele** | **MAF (cases)** | **MAF (controls)** | **HWE** **ALL** | **HWE**  **AFF** | **HWE**  **UNAFF** |
| --- | --- | --- | --- | --- | --- | --- | --- | --- | --- | --- | --- |
| ***PNPLA1*** | 6p21.31 | rs13196184 | 36314060 | Near | 428/457 | T | 0.071 | 0.067 | 0.062 | 0.271 | 0.114 |
|  | 6p21.31 | rs9380559 | 36315576 | Near | 452/484 | G | 0.43 | 0.42 | 0.105 | 0.269 | 0.206 |
|  | 6p21.31 | rs4395718 | 36323671 | Intronic | 452/483 | A | 0.22 | 0.22 | 0.349 | 0.083 | 0.692 |
|  | 6p21.31 | rs12196677 | 36334503 | Intronic | 451/480 | G | 0.083 | 0.085 | 0.205 | 0.360 | 0.373 |
|  | 6p21.31 | rs7759056 | 36334614 | Intronic | 446/476 | A | 0.16 | 0.17 | 0.105 | 0.162 | 0.437 |
|  | 6p21.31 | rs7775107 | 36337473 | Intronic | 416/476 | A | 0.40 | 0.41 | 0.022 | 0.057 | 0.183 |
|  | 6p21.31 | rs4713944 | 36338113 | Intronic | 454/484 | G | 0.48 | 0.46 | 0.799 | 0.785 | 1.000 |
|  | 6p21.31 | rs11753002 | 36342154 | Intronic | 460/485 | C | 0.11 | 0.098 | 0.599 | 0.635 | 1.000 |
|  | 6p21.31 | rs12202603 | 36351472 | Intronic | 444/451 | G | 0.27 | 0.29 | 0.427 | 0.343 | 0.831 |
|  | 6p21.31 | rs1547421 | 36354548 | Intronic | 457/485 | T | 0.39 | 0.40 | 0.208 | 0.216 | 0.581 |
|  | 6p21.31 | rs12526665 | 36354685 | Intronic | 455/482 | A | 0.17 | 0.14 | 0.389 | 0.629 | 0.566 |
|  | 6p21.31 | rs12212459 | 36360182 | Intronic | 441/464 | C | 0.41 | 0.41 | 0.947 | 1.000 | 1.000 |
|  | 6p21.31 | rs1467912 | 36361188 | Intronic | 456/482 | T | 0.37 | 0.39 | 0.947 | 1.000 | 1.000 |
|  | 6p21.31 | rs2239795 | 36368836 | Exon 3 | 444/473 | T | 0.36 | 0.37 | 0.145 | 0.424 | 0.211 |
|  | 6p21.31 | rs2239796 | 36368892 | Intronic | 452/478 | T | 0.32 | 0.32 | 0.274 | 0.295 | 0.684 |
|  | 6p21.31 | rs17356524 | 36370905 | Intronic | 454/485 | T | 0.12 | 0.12 | 0.769 | 0.831 | 0.413 |
|  | 6p21.31 | rs4713951 | 36370969 | Intronic | 452/479 | T | 0.46 | 0.47 | 0.848 | 0.854 | 0.657 |
|  | 6p21.31 | rs732394 | 36377062 | Intronic | 445/464 | C | 0.47 | 0.48 | 0.196 | 0.402 | 0.325 |
|  | 6p21.31 | rs10947600 | 36377581 | Intronic | 457/482 | A | 0.41 | 0.41 | 0.358 | 0.851 | 0.236 |
|  | 6p21.31 | rs12199580 | 36378108 | Exon 6 | 452/485 | A | 0.41 | 0.42 | 0.238 | 0.573 | 0.274 |
|  | 6p21.31 | rs4713955 | 36378646 | Intronic | 437/456 | A | 0.24 | 0.22 | 0.010 | 0.056 | 0.088 |
|  | 6p21.31 | rs12189786 | 36380018 | Intronic | 451/477 | G | 0.16 | 0.16 | 0.906 | 0.865 | 0.623 |
|  | 6p21.31 | rs12197079 | 36382131 | Exon 7 | 446/472 | T | 0.27 | 0.30 | 0.581 | 0.907 | 0.336 |
|  | 6p21.31 | rs4713956 | 36383436 | Exon 8 | 457/485 | T | 0.37 | 0.33 | 0.781 | 0.080 | 0.164 |
|  | 6p21.31 | rs12662591 | 36384788 | Locus | 460/484 | A | 0.14 | 0.15 | 0.702 | 0.123 | 0.395 |
|  | 6p21.31 | rs7738417 | 36385529 | Near | 456/482 | A | 0.29 | 0.31 | 0.544 | 0.911 | 0.534 |
| ***PNPLA2*** | 11p15.5 | rs7942159 | 812622 | Intronic | 452/477 | A | 0.42 | 0.38 | 0.549 | 0.778 | 0.637 |
|  | 11p15.5 | rs1138693 | 814789 | Exon 10 | 440/477 | T | 0.28 | 0.26 | 0.014 | 0.013 | 0.345 |
|  | 11p15.5 | rs7126805 | 818916 | Near* | 454/482 | G | 0.29 | 0.28 | 0.876 | 0.911 | 0.913 |
| ***PNPLA3*** | 22q13.31 | rs929092 | 42641985 | Near | 459/486 | G | 0.44 | 0.46 | 0.848 | 0.783 | 1.000 |
|  | 22q13.31 | rs4823104 | 42646050 | Intronic | 453/479 | G | 0.088 | 0.092 | 0.239 | 0.243 | 0.597 |
|  | 22q13.31 | rs2076213 | 42647823 | Exon 2 | 450/478 | G | 0.11 | 0.10 | 0.323 | 0.814 | 0.174 |
|  | 22q13.31 | rs2076212 | 42647871 | Exon 2 | 462/484 | T | 0.13 | 0.11 | 0.374 | 0.071 | 0.660 |
|  | 22q13.31 | rs139047 | 42647975 | Intronic | 453/481 | A | 0.41 | 0.44 | 0.153 | 0.047 | 0.929 |
|  | 22q13.31 | rs9625961 | 42648120 | Intronic | 455/479 | G | 0.16 | 0.15 | 0.405 | 0.406 | 0.865 |
|  | 22q13.31 | rs738407 | 42648856 | Intronic | 449/467 | C | 0.38 | 0.41 | 0.381 | 0.203 | 1.000 |
|  | 22q13.31 | rs2006943 | 42649082 | Intronic | 452/486 | A | 0.24 | 0.25 | 0.194 | 0.263 | 0.547 |
|  | 22q13.31 | rs139051 | 42649577 | Intronic | 454/469 | A | 0.35 | 0.40 | 0.632 | 0.187 | 0.711 |
|  | 22q13.31 | rs738409 | 42649628 | Exon 3 | 461/486 | G | 0.22 | 0.25 | 0.065 | 0.510 | 0.061 |
|  | 22q13.31 | rs12483959 | 42650897 | Intronic | 451/484 | A | 0.16 | 0.20 | 0.063 | 0.609 | 0.064 |
|  | 22q13.31 | rs9626056 | 42651976 | Intronic | 454/486 | T | 0.061 | 0.044 | 0.193 | 0.693 | 0.083 |
|  | 22q13.31 | rs1883350 | 42652944 | Intronic | 458/486 | C | 0.32 | 0.34 | 0.720 | 0.253 | 0.552 |
|  | 22q13.31 | rs2072907 | 42657554 | Intronic | 456/477 | C | 0.17 | 0.21 | 0.051 | 0.422 | 0.082 |
|  | 22q13.31 | rs3810622 | 42663035 | Intronic | 442/467 | C | 0.43 | 0.39 | 0.028 | 0.109 | 0.186 |
|  | 22q13.31 | rs2294917 | 42666887 | Intronic | 457/477 | C | 0.35 | 0.33 | 0.202 | 0.191 | 0.688 |
|  | 22q13.31 | rs2294918 | 42667017 | Exon 9 | 451/483 | A | 0.39 | 0.39 | 0.789 | 0.503 | 0.852 |
|  | 22q13.31 | rs2294919 | 42667226 | 3´UTR | 454/482 | T | 0.22 | 0.23 | 0.927 | 0.792 | 0.614 |
| ***PNPLA4*** | Xp22.3 | rs6639973 | 7673755 | Near | 452/480 | G | 0.27 | 0.25 | 0.390 | 0.531 | 0.074 |
|  | Xp22.3 | rs17310972 | 7680753 | Intronic | 450/480 | G | 0.092 | 0.11 | 0.012 | 0.090 | 0.060 |
|  | Xp22.3 | rs1179136 | 7694616 | Intronic | 459/485 | T | 0.13 | 0.11 | 0.523 | 0.143 | 0.529 |
|  | Xp22.3 | rs1179131 | 7706012 | Near | 451/485 | C | 0.45 | 0.47 | 0.931 | 0.614 | 0.557 |
| ***PNPLA5*** | 22q13.31 | rs2213725 | 42599015 | Near | 456/481 | A | 0.085 | 0.080 | 0.830 | 0.141 | 0.347 |
|  | 22q13.31 | rs470093 | 42601072 | 3´UTR | 454/477 | T | 0.14 | 0.13 | 0.602 | 0.854 | 0.272 |
|  | 22q13.31 | rs12485136 | 42601445 | 3´UTR | 449/477 | A | 0.10 | 0.11 | 0.235 | 0.075 | 1.000 |
|  | 22q13.31 | rs739231 | 42607177 | Exon 6 | 458/486 | G | 0.40 | 0.39 | 0.509 | 0.925 | 0.461 |
|  | 22q13.31 | rs9626043 | 42609716 | Intronic | 459/486 | G | 0.16 | 0.18 | 0.655 | 0.490 | 1.000 |
|  | 22q13.31 | rs2401203 | 42609755 | Intronic | 451/473 | A | 0.51 | 0.49 | 0.225 | 0.927 | 0.110 |
|  | 22q13.31 | rs2071883 | 42611851 | Exon 2 | 453/480 | A | 0.13 | 0.11 | 0.769 | 0.845 | 0.824 |
|  | 22q13.31 | rs916358 | 42612108 | Intronic | 459/484 | T | 0.13 | 0.11 | 0.884 | 0.846 | 0.829 |
|  | 22q13.31 | rs763118 | 42616200 | Near | 451/484 | G | 0.24 | 0.25 | 0.796 | 0.902 | 0.904 |
|  | 22q13.31 | rs5764391 | 42616559 | Near | 448/474 | T | 0.093 | 0.089 | 0.030 | 0.164 | 0.095 |

PNPLA - patatin-like phospholipase, CHR – Chromosome, SNP – Singel nucleotide polymorphism, BP – base pair position, HWE – Hardy Weinberg equilibrium, Aff – obese cases, UNAFF – non-obese controls, rs-id in bold indicates coding SNPs, * located in exon 3 in the nearby gene *EFCAB4A*.
