# Supplementary material for: Genetic Variance in the Adiponutrin Gene Family and Childhood Obesity
Source: PLoS One. 2009 Apr 24;4(4):e5327. doi: 10.1371/journal.pone.0005327 (PMC2669125; doi:10.1371/journal.pone.0005327)
Supplement: Table S2 — Variants in the five genes in the Adiponutrin gene family and the association with obesity using logistic regression analyses. (0.12 MB DOC) [file pone.0005327.s002.doc]

***Supporting Table S2:*** Variants in the five genes in the Adiponutrin gene family and the association with obesity using logistic regression analyses.

| **Gene** | **CHR** | **SNP** | **N(case/control)** | **OR** | **95% CI** | ***P*** | ***P*(age,gender)** |
| --- | --- | --- | --- | --- | --- | --- | --- |
| *PNPLA1* | 6 | rs13196184 | 884 | 1.48 | (0.79-2.77) | 0.72 | 0.23 |
|  | 6 | rs9380559 | 935 | 1.42 | (1.02-1.98) | 0.63 | 0.038 |
|  | 6 | rs4395718 | 934 | 1.10 | (0.74-1.63) | 0.89 | 0.64 |
|  | 6 | rs12196677 | 930 | 1.23 | (0.70-2.14) | 0.88 | 0.48 |
|  | 6 | rs7759056 | 921 | 1.24 | (0.80-1.92) | 0.40 | 0.33 |
|  | 6 | rs7775107 | 891 | 1.16 | (0.84-1.59) | 0.68 | 0.36 |
|  | 6 | rs4713944 | 937 | 1.05 | (0.76-1.44) | 0.30 | 0.79 |
|  | 6 | rs11753002 | 944 | 1.14 | (0.67-1.92) | 0.59 | 0.63 |
|  | 6 | rs12202603 | 894 | 0.79 | (0.55-1.14) | 0.28 | 0.21 |
|  | 6 | rs1547421 | 941 | 0.78 | (0.56-1.10) | 0.66 | 0.16 |
|  | 6 | rs12526665 | 936 | 1.32 | (0.84-2.08) | 0.063 | 0.23 |
|  | 6 | rs12212459 | 904 | 0.70 | (0.50-0.99) | 0.94 | 0.043 |
|  | 6 | rs1467912 | 937 | 0.71 | (0.51-1.00) | 0.61 | 0.049 |
|  | 6 | rs2239795 | 916 | 1.32 | (0.94-1.86) | 0.64 | 0.11 |
|  | 6 | rs2239796 | 929 | 0.81 | (0.57-1.15) | 0.82 | 0.24 |
|  | 6 | rs17356524 | 938 | 0.72 | (0.44-1.18) | 0.91 | 0.19 |
|  | 6 | rs4713951 | 930 | 0.71 | (0.51-0.98) | 0.67 | 0.037 |
|  | 6 | rs732394 | 908 | 0.74 | (0.54-1.02) | 0.89 | 0.067 |
|  | 6 | rs10947600 | 938 | 0.72 | (0.52-0.99) | 0.87 | 0.042 |
|  | 6 | rs12199580 | 936 | 0.70 | (0.51-0.96) | 0.64 | 0.028 |
|  | 6 | rs4713955 | 892 | 0.98 | (0.68-1.40) | 0.33 | 0.89 |
|  | 6 | rs12189786 | 927 | 1.08 | (0.71-1.63) | 0.93 | 0.74 |
|  | 6 | rs12197079 | 917 | 1.02 | (0.71-1.45) | 0.21 | 0.93 |
|  | 6 | rs4713956 | 941 | 0.94 | (0.68-1.30) | 0.065 | 0.71 |
|  | 6 | rs12662591 | 943 | 1.00 | (0.63-1.57) | 0.44 | 0.98 |
|  | 6 | rs7738417 | 937 | 1.08 | (0.76-1.54) | 0.33 | 0.68 |
| *PNPLA2* | 11 | rs7942159 | 928 | 1.27 | (0.91-1.76) | 0.12 | 0.16 |
|  | 11 | rs1138693 | 916 | 1.20 | (0.85-1.70) | 0.39 | 0.31 |
|  | 11 | rs7126805 | 935 | 0.93 | (0.66-1.31) | 0.88 | 0.69 |
| *PNPLA3* | 22 | rs929092 | 944 | 0.97 | (0.71-1.34) | 0.37 | 0.87 |
|  | 22 | rs4823104 | 931 | 1.03 | (0.61-1.76) | 0.77 | 0.91 |
|  | 22 | rs2076213 | 927 | 1.11 | (0.66-1.87) | 0.76 | 0.69 |
|  | 22 | rs2076212 | 945 | 1.12 | (0.70-1.78) | 0.17 | 0.65 |
|  | 22 | rs139047 | 933 | 0.78 | (0.57-1.05) | 0.19 | 0.10 |
|  | 22 | rs9625961 | 933 | 1.13 | (0.73-1.77) | 0.32 | 0.58 |
|  | 22 | rs738407 | 916 | 0.95 | (0.68-1.33) | 0.20 | 0.77 |
|  | 22 | rs2006943 | 937 | 1.04 | (0.72-1.51) | 0.64 | 0.84 |
|  | 22 | rs139051 | 922 | 0.99 | (0.71-1.38) | 0.014 | 0.97 |
|  | 22 | rs738409 | 946 | 0.89 | (0.61-1.30) | 0.13 | 0.55 |
|  | 22 | rs12483959 | 934 | 0.79 | (0.52-1.20) | 0.023 | 0.27 |
|  | 22 | rs9626056 | 939 | 1.33 | (0.66-2.66) | 0.12 | 0.42 |
|  | 22 | rs1883350 | 943 | 0.96 | (0.68-1.36) | 0.39 | 0.83 |
|  | 22 | rs2072907 | 932 | 0.82 | (0.55-1.24) | 0.041 | 0.35 |
|  | 22 | rs3810622 | 908 | 1.28 | (0.94-1.75) | 0.063 | 0.12 |
|  | 22 | rs2294917 | 933 | 1.25 | (0.90-1.74) | 0.38 | 0.18 |
|  | 22 | rs2294918 | 933 | 0.90 | (0.65-1.25) | 0.92 | 0.54 |
|  | 22 | rs2294919 | 935 | 1.11 | (0.76-1.62) | 0.72 | 0.59 |
| *PNPLA4** | 23 | rs6639973 | 930 | 1.00 | (0.66-1.51) | 0.30 | 0.99 |
|  | 23 | rs17310972 | 926 | 1.00 | (0.51-1.95) | 0.30 | 1.00 |
|  | 23 | rs1179136 | 943 | 0.89 | (0.50-1.59) | 0.23 | 0.69 |
|  | 23 | rs1179131 | 933 | 1.15 | (0.79-1.68) | 0.59 | 0.47 |
| *PNPLA5* | 22 | rs2213725 | 936 | 1.10 | (0.62-1.96) | 0.68 | 0.74 |
|  | 22 | rs470093 | 930 | 1.29 | (0.81-2.07) | 0.36 | 0.29 |
|  | 22 | rs12485136 | 925 | 0.80 | (0.49-1.29) | 0.56 | 0.36 |
|  | 22 | rs739231 | 943 | 1.16 | (0.83-1.62) | 0.74 | 0.39 |
|  | 22 | rs9626043 | 944 | 1.03 | (0.66-1.59) | 0.27 | 0.91 |
|  | 22 | rs2401203 | 923 | 1.03 | (0.74-1.43) | 0.37 | 0.88 |
|  | 22 | rs2071883 | 932 | 1.16 | (0.70-1.93) | 0.15 | 0.57 |
|  | 22 | rs916358 | 942 | 1.03 | (0.63-1.70) | 0.19 | 0.90 |
|  | 22 | rs763118 | 934 | 0.83 | (0.58-1.19) | 0.78 | 0.31 |
|  | 22 | rs5764391 | 921 | 1.13 | (0.66-1.92) | 0.80 | 0.66 |

*p*-values are calculated using Logistic regression including gender and age as covariates, additive model. The presented Odds ratios (OR) are adjusted for age and gender. SNP – Single nucleotide polymorphism, BP – base pair position, A1 – minor allele, Non missing – Number of individuals with genotype data of the total 1015 children, OR – Odds ratio, CI 95% - 95% confidence interval. *The unadjusted *p*-value is adjusted for gender since *PNPLA4* is located on the X-chromosome.
