# Supplementary figures and images for: Genetic Variance in the Adiponutrin Gene Family and Childhood Obesity
Source: PLoS One. 2009 Apr 24;4(4):e5327. doi: 10.1371/journal.pone.0005327 (PMC2669125; doi:10.1371/journal.pone.0005327)

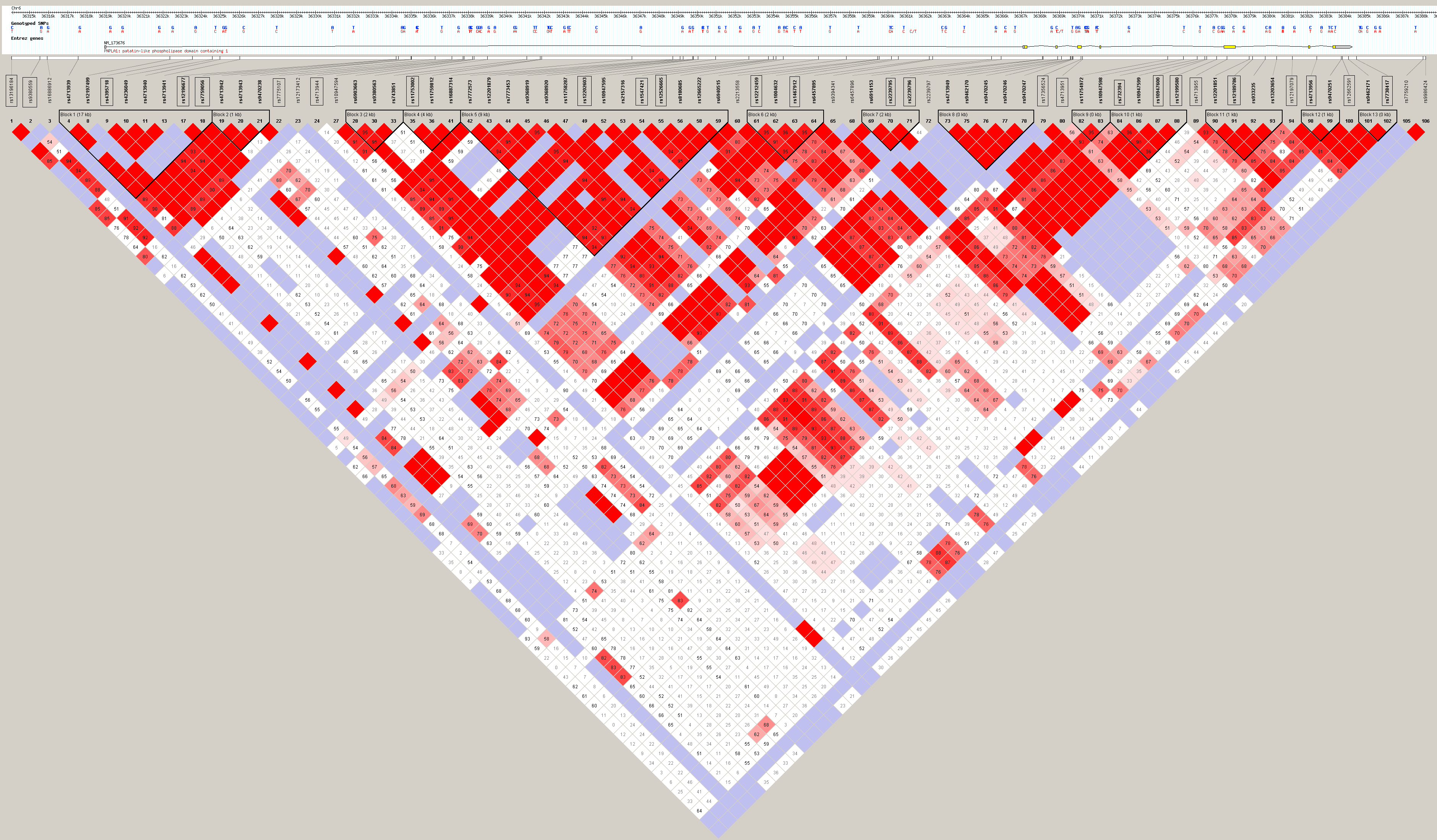

Supplement: Figure S1 — Graphical overview of the patatin-like phospholipase 1 (PNPLA1) gene and linkage disequilibrium obtained from HapMap (http://www.hapmap.org/). SNPs successfully genotyped by MALDI-TOF MS are marked with a black square. (0.62 MB PNG) [file pone.0005327.s003.png]

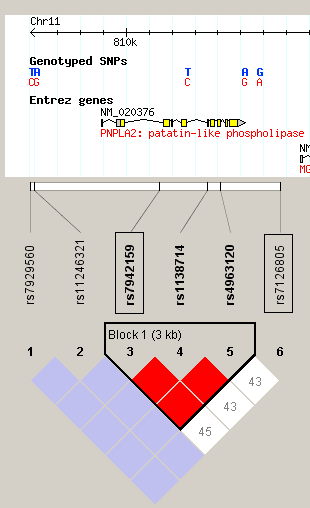

Supplement: Figure S2 — Graphical overview of the patatin-like phospholipase 2 (PNPLA2) gene and linkage disequilibrium obtained from HapMap (http://www.hapmap.org/). SNPs successfully genotyped by MALDI-TOF MS are marked with a black square. One SNP, the rs1138693, is not included since it was not present in the HapMap database at the time of data extraction. It was included in the study because it is a coding SNP. (0.01 MB PNG) [file pone.0005327.s004.png]

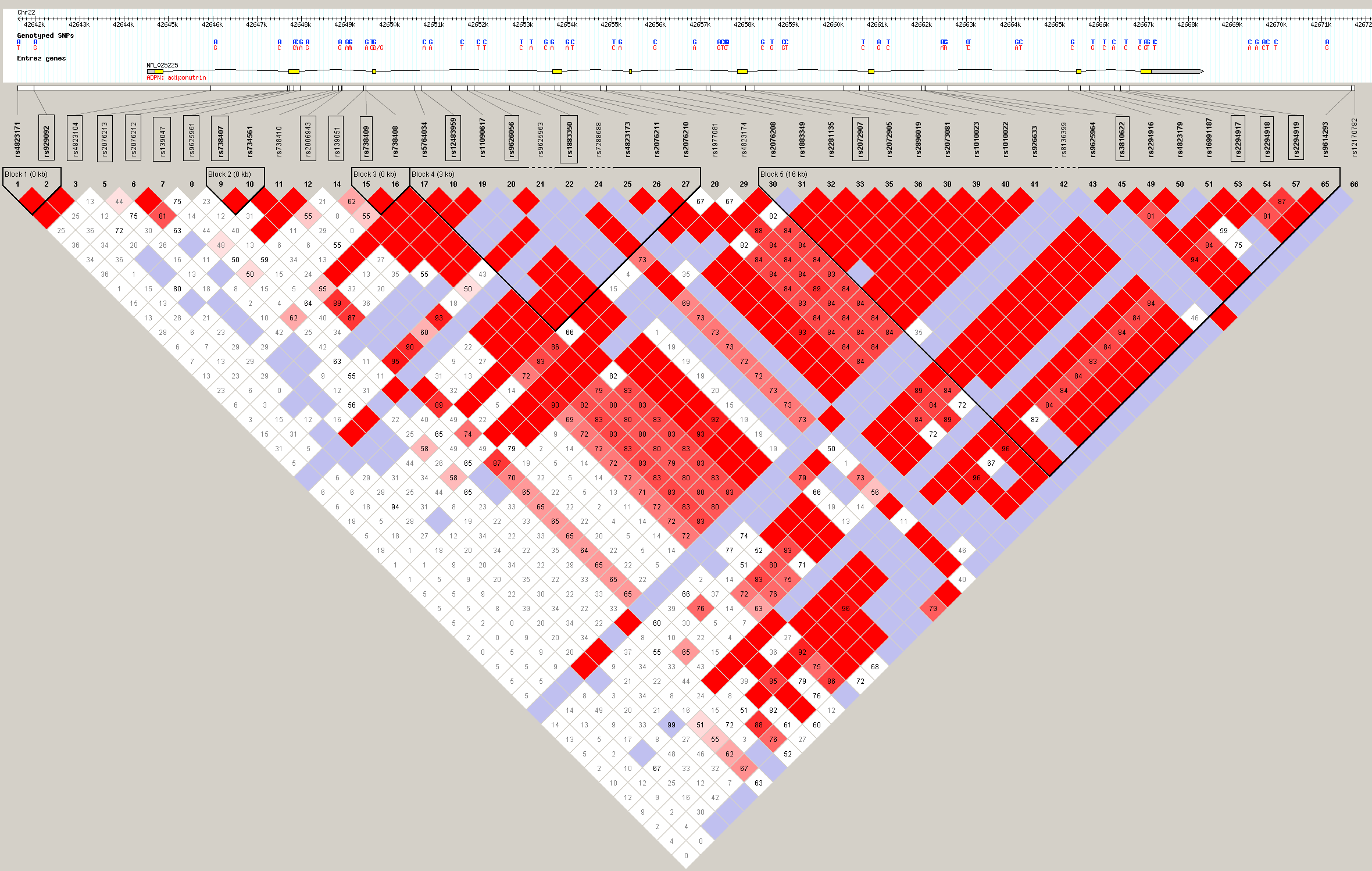

Supplement: Figure S3 — Graphical overview of the patatin-like phospholipase 3 (PNPLA3) gene and linkage disequilibrium obtained from HapMap (http://www.hapmap.org/). SNPs successfully genotyped by MALDI-TOF MS are marked with a black square. (0.26 MB PNG) [file pone.0005327.s005.png]

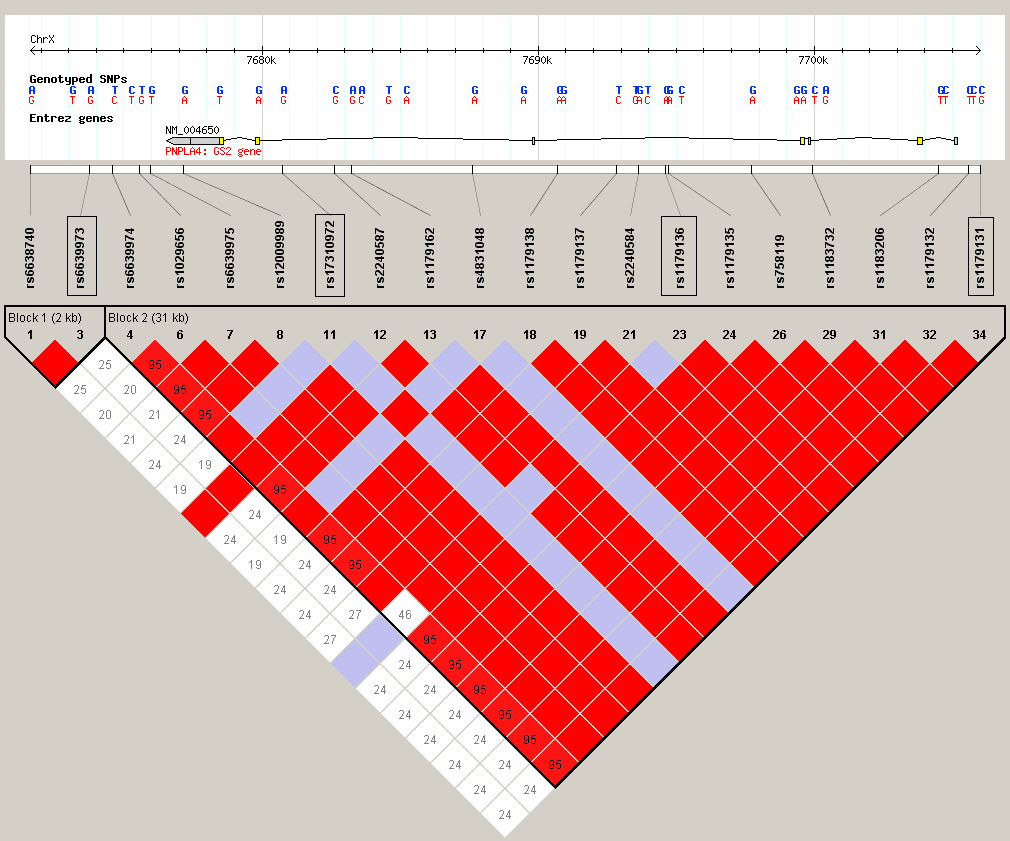

Supplement: Figure S4 — Graphical overview of the patatin-like phospholipase 4 (PNPLA4) gene and linkage disequilibrium obtained from HapMap (http://www.hapmap.org/). SNPs successfully genotyped by MALDI-TOF MS are marked with a black square. (0.06 MB PNG) [file pone.0005327.s006.png]

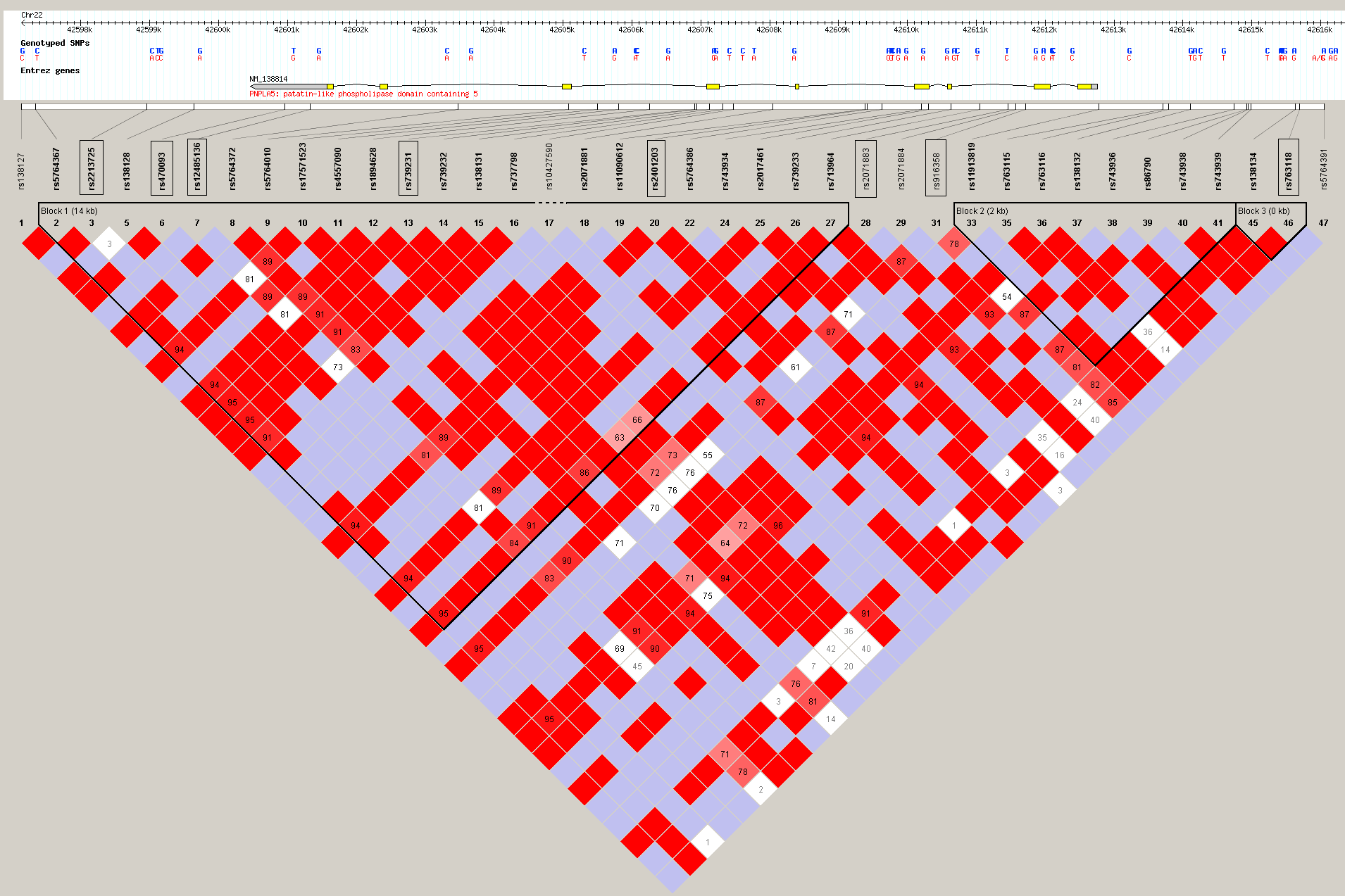

Supplement: Figure S5 — Graphical overview of the patatin-like phospholipase 5 (PNPLA5) gene and linkage disequilibrium obtained from HapMap (http://www.hapmap.org/). SNPs successfully genotyped by MALDI-TOF MS are marked with a black square. (0.17 MB PNG) [file pone.0005327.s007.png]
